# Lysine 27 of replication-independent histone H3.3 is required for Polycomb target gene silencing but not for gene activation

**DOI:** 10.1371/journal.pgen.1007932

**Published:** 2019-01-30

**Authors:** Mary Leatham-Jensen, Christopher M. Uyehara, Brian D. Strahl, A. Gregory Matera, Robert J. Duronio, Daniel J. McKay

**Affiliations:** 1 Department of Biology, The University of North Carolina at Chapel Hill, Chapel Hill, NC, United States of America; 2 Department of Genetics, The University of North Carolina at Chapel Hill, Chapel Hill, NC, United States of America; 3 Integrative Program for Biological and Genome Sciences, The University of North Carolina at Chapel Hill, Chapel Hill, NC, United States of America; 4 Curriculum in Genetics and Molecular Biology, The University of North Carolina at Chapel Hill, Chapel Hill, NC, United States of America; 5 Department of Biochemistry and Biophysics, The University of North Carolina at Chapel Hill, Chapel Hill, NC, United States of America; 6 Lineberger Comprehensive Cancer Center, The University of North Carolina at Chapel Hill, Chapel Hill, NC, United States of America; Stanford University School of Medicine, UNITED STATES

## Abstract

Proper determination of cell fates depends on epigenetic information that is used to preserve memory of decisions made earlier in development. Post-translational modification of histone residues is thought to be a central means by which epigenetic information is propagated. In particular, modifications of histone H3 lysine 27 (H3K27) are strongly correlated with both gene activation and gene repression. H3K27 acetylation is found at sites of active transcription, whereas H3K27 methylation is found at loci silenced by Polycomb group proteins. The histones bearing these modifications are encoded by the replication-dependent H3 genes as well as the replication-independent H3.3 genes. Owing to differential rates of nucleosome turnover, H3K27 acetylation is enriched on replication-independent H3.3 histones at active gene loci, and H3K27 methylation is enriched on replication-dependent H3 histones across silenced gene loci. Previously, we found that modification of replication-dependent H3K27 is required for Polycomb target gene silencing, but it is not required for gene activation. However, the contribution of replication-independent H3.3K27 to these functions is unknown. Here, we used CRISPR/Cas9 to mutate the endogenous replication-independent H3.3K27 to a non-modifiable residue. Surprisingly, we find that H3.3K27 is also required for Polycomb target gene silencing despite the association of H3.3 with active transcription. However, the requirement for H3.3K27 comes at a later stage of development than that found for replication-dependent H3K27, suggesting a greater reliance on replication-independent H3.3K27 in post-mitotic cells. Notably, we find no evidence of global transcriptional defects in H3.3K27 mutants, despite the strong correlation between H3.3K27 acetylation and active transcription.

## Author summary

During development, naïve precursor cells acquire distinct identities through differential regulation of gene expression. The process of cell fate specification is progressive and depends on memory of prior developmental decisions. Maintaining cell identities over time is not dependent on changes in genome sequence. Instead, epigenetic mechanisms propagate information on cell identity by maintaining select sets of genes in either the on or off state. Chemical modifications of histone proteins, which package and organize the genome within cells, are thought to play a central role in epigenetic gene regulation. However, identifying which histone modifications are required for gene regulation, and defining the mechanisms through which they function in the maintenance of cell identity, remains a longstanding research challenge. Here, we focus on the role of histone H3 lysine 27 (H3K27). Modifications of H3K27 are associated with both gene activation and gene silencing (i.e. H3K27 acetylation and methylation, respectively). The histones bearing these modifications are encoded by different histone genes. One set of histone genes is only expressed during cell division, whereas the other set of histone genes is expressed in both dividing and non-dividing cells. Because most cells permanently stop dividing by the end of development, these “replication-independent” histone genes are potentially important for long-term maintenance of cell identity. In this study, we demonstrate that replication-independent H3K27 is required for gene silencing by the Polycomb group of epigenetic regulators. However, despite a strong correlation between replication-independent histones and active genes, we find that replication-independent H3K27 is not required for gene activation. As mutations in replication-independent H3K27 have recently been identified in human cancers, this work may help to inform the mechanisms by which histone mutations contribute to human disease.

## Introduction

Inside the nucleus, DNA is wrapped around histone proteins to form nucleosomes, the basic building block of chromatin. In addition to compacting the genome within the confines of a small nuclear space, histone proteins also serve as substrates for regulating genome activity. In particular, many DNA-dependent processes are regulated by histone post-translational modifications (PTMs). Histone PTMs function primarily by controlling the assembly of regulatory complexes at specific loci in the genome, either through direct alteration of chromatin structure, or by serving as binding sites for *trans*-acting factors [1]. Due to the inheritance of parental histones by daughter cells during cell division, histone PTMs also have the potential to propagate epigenetic information over time, thereby helping to maintain the gene expression programs underlying cell identity. A longstanding objective has been to identify histone PTMs involved in gene regulation and to determine their relative importance in maintenance of cell identity.

A wide range of histone PTMs have been described, including acetylation, methylation, and ubiquitylation [1, 2]. Many of these PTMs are enriched at particular types of functional elements in the genome. For example, tri-methylation of H3K36 is enriched at gene exons [3], whereas H3K9 tri-methylation is enriched in heterochromatin [4]. While the presence of histone PTMs at particular DNA elements genome wide suggests a role in regulating the activity of these elements, the specific function of histone PTMs remains largely untested in animals. This is because PTM function has been inferred from mutations in the proteins that catalyze or bind to PTMs. However, the “writer” and “reader” proteins for histone PTMs typically belong to multi-protein complexes with numerous regulatory targets, making interpretation of their mutant phenotypes challenging. For example, ubiquitylation of histone H2A is strongly correlated with transcriptional silencing by Polycomb group proteins. Moreover, null mutations in *Drosophila* Sce or its mammalian homologs Ring1A/B [5] [6] [7], which catalyze this PTM, result in de-repression of Polycomb target genes, leading to the conclusion that H2A ubiquitylation plays a critical role in Polycomb target gene silencing. However, more recently, mutation of the lysine residues in H2A that prevent its ubiquitylation by Sce did not result in Polycomb target gene de-repression [8], indicating that H2A ubiquitylation is dispensable for Polycomb-mediating gene silencing in *Drosophila*. Similar findings have been described for histone H3 lysine 4 (H3K4), whose modification is associated with transcriptional activation. Mono-methylation of H3K4 is found at transcriptional enhancers and tri-methylation of H3K4 is correlated with active promoters [9]. However, mutation of H3K4 in *Drosophila* revealed that its modification is not required for signal transduction-mediated gene expression [10]. Thus, mutating histone residues is an effective means of determining the requirement of PTMs in regulating genome activity.

In this study, we examine the role of histone H3 lysine 27 (H3K27) in transcriptional regulation. Modification of H3K27 is correlated with both gene activation and gene repression. H3K27 acetylation (H3K27ac), which is catalyzed by the histone acetyltransferase CBP/p300 [11], is associated with gene activation. Although H3K4 mono-methylation is found at DNA elements that have the potential for enhancer activity (ie. poised enhancers) [12], the appearance of H3K27ac coincides with enhancer activation [13]. The strong correlation between H3K27ac and enhancer activity has led it to become a defining feature of active enhancers [14]. Consistent with an important role of H3K27ac in enhancer function, mutations in the genes encoding CBP/p300 are lethal [15]. Despite its prominence, the precise role of H3K27ac in enhancer activation remains unclear. In contrast to H3K27ac, methylation of H3K27 is associated with gene repression. H3K27 tri-methylation (H3K27me3) in particular is closely correlated with transcriptional silencing by Polycomb group complexes [16]. In *Drosophila*, H3K27me3 is catalyzed by the methyltransferase Enhancer of Zeste (E(z)), a core component of the Polycomb Repressive Complex 2 (PRC2) [17–19]. H3K27me3 is specifically bound by the chromodomain of the Polycomb (Pc) protein, a core component of the PRC1 complex [20, 21]. Together, PRC1 and PRC2, as well as additional Polycomb repressive complexes, maintain heritable silencing of target genes during development. Mutations in *E(z)* or *Pc* result in transcriptional de-repression of Polycomb targets such as the Hox factors and other master regulators of cell identity [16, 18]. In mammals, mutations in Polycomb group proteins are associated with oncogenesis and defects in stem cell function [22], making understanding of Polycomb function important for human health. However, the mechanisms whereby H3K27 modifications influence maintenance of transcriptional “on” and “off” states remain elusive.

In dividing cells, the majority of histones are packaged into chromatin during DNA replication. These histones are encoded by the replication-dependent histone genes, which are expressed at high levels only during S-phase [23]. Outside of S-phase, replacement of histones disrupted by DNA-dependent processes is accomplished by the replication-independent histone genes, which are expressed throughout the cell cycle [24]. Remarkably, in post-mitotic cells like neurons, replication-independent histones accumulate with age to near-saturating levels [25] [26]. In *Drosophila*, the replication-dependent histone H3 is termed H3.2, and the replication-independent histone H3 is termed H3.3. Genome-wide profiling has revealed that H3.3 is enriched at sites of nucleosome turnover [27] [28] [29]. These are sites where nucleosomes have been replaced due to disruption of histone-DNA interactions, such as within the bodies of actively transcribed genes, as well as in enhancers and promoters. Consistent with the high levels of H3.3 at these loci, mass spectrometry indicates that H3.3 is enriched relative to H3.2 for histone PTMs associated with active chromatin, including H3K4 methylation and H3K27ac, in both fly and human cells [30] [31]. H3.3 is also enriched at other sites of nucleosome turnover, including those associated with gene repression such as Polycomb Response Elements (PREs) [27].

The role of H3.2K27 was recently tested by genetic replacement of the endogenous replication-dependent histone genes with transgenes encoding mutant histones that cannot be modified on lysine 27 (H3.2K27R) [32] [33]. This work revealed that H3.2K27 is required for Polycomb target gene silencing but not for gene activation. Mutations in replication-independent H3.3K27 have been identified in pediatric cancer patients. In particular, a recurrent H3.3K27M mutation was found in 32/40 cases (80%) of diffuse intrinsic pontine gliomas [34]. These cancers also exhibit global decreases in H3K27me3 levels and an increase in H3K27ac [35]. Mechanistically, it has been proposed that H3.3K27M drives oncogenesis by dominantly interfering with PRC2 activity [35]. In *Drosophila*, transgenic overexpression of H3.3K27M phenocopies loss of function mutations in Polycomb group proteins [36], suggesting a conserved mechanism of action. However, despite this work, the role played by H3.3K27 in normal development has not been described.

In this report, we use CRISPR/Cas9 to mutate lysine 27 of the replication-independent H3.3 genes in *Drosophila* to a non-modifiable residue (H3.3K27R). Surprisingly, despite loss of H3.3K27ac, we observe no global defects in steady-state RNA levels in H3.3K27R mutants, indicating that H3.3K27ac is dispensable for transcription. However, Polycomb group target genes become de-repressed at late stages of development in H3.3K27R mutants, indicating that H3.3K27 is required for proper long-term epigenetic silencing by Polycomb.

## Results

### H3.3B^K27R^ causes male lethality

**Fig 1:**
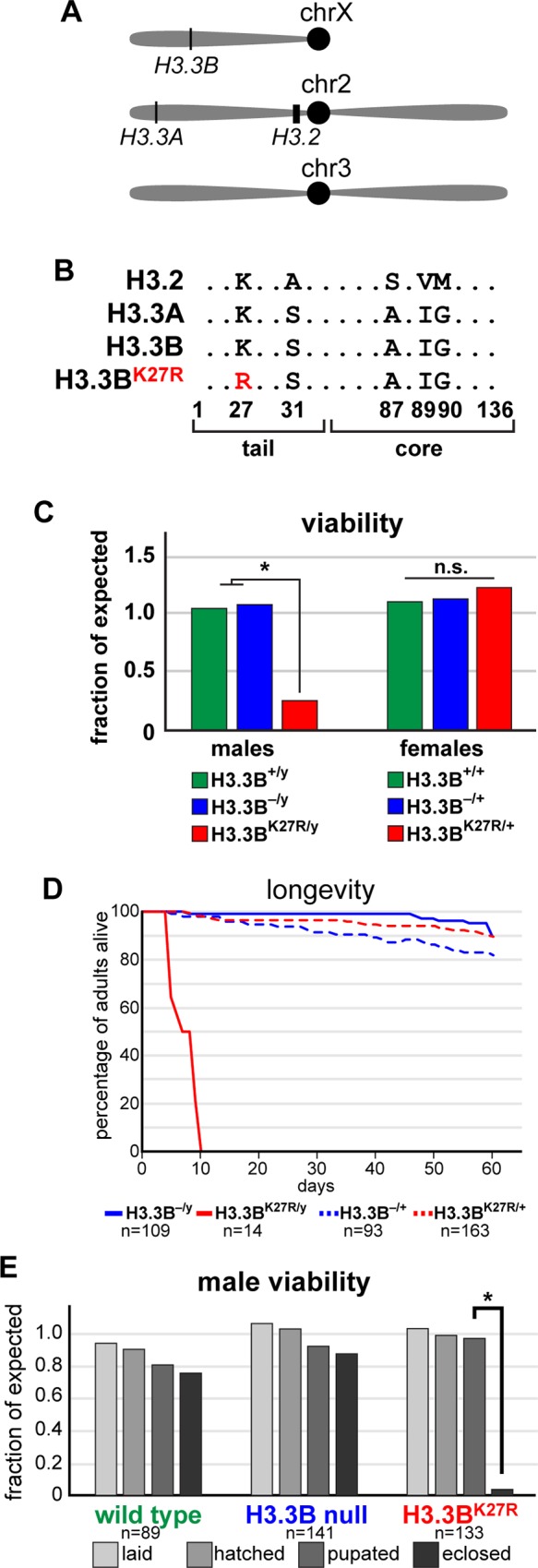
*H3*.*3B*^*K27R*^ causes male lethality. (**A**) Cartoon depicting the genomic locations of the three H3 loci. (**B**) Amino acid sequence of the three H3 proteins and the H3.3^K27R^ mutant (highlighted in red). (**C**) Bar plot of the viability of wild type (green) males and females, as well as hemizygous males and heterozygous females for an *H3*.*3B* null allele (blue), and for the *H3*.*3B*^*K27R*^ allele (red). Data are plotted as the fraction of expected based on the total number of flies. Asterisk indicates fewer than expected males survive (chi-square test, p value < 0.001). (**D**) Kaplan-Meyer plot of hemizygous males (solid lines) and heterozygous females (dashed lines) for *H3*.*3B* null (blue) and *H3*.*3B*^*K27R*^ (red) alleles. n equals the number of adults assayed. (**E**) Bar plot of male viability at four developmental stages for the three indicated genotypes. Asterisk indicates fewer than expected males survive the pupal stage (chi-square test, p value < 0.001). n equals the starting number of eggs for each genotype.

Similar to mammalian genomes, the *Drosophila* genome contains two H3.3 genes that encode proteins with identical sequences [37]. *H3*.*3A* is located on the second chromosome, and *H3*.*3B* is located on the X chromosome (**Fig 1A**). Null mutations in either gene individually result in no mutant phenotype, suggesting functional overlap between H3.3A and H3.3B. By contrast, H3.3 double mutants exhibit reduced viability and are infertile [38, 39]. The H3.3 proteins differ from the replication-dependent H3.2 by four amino acids (**Fig 1B**). Three of these four residues play an important role in chaperone-dependent deposition of H3.3 into chromatin outside of S-phase [24], resulting in distinct genome-wide distribution profiles for H3.3 and H3.2. Aside from these four amino acids, the H3.2 and H3.3 protein sequences are identical, suggesting they may perform largely similar functions in chromatin. Indeed, H3.2 rescues the reduced viability of H3.3 double mutants when expressed under control of H3.3 *cis*-regulatory sequences [39]. Conversely, H3.3 rescues lethality of replication-dependent histone mutants when expressed under control of H3.2 *cis*-regulatory sequences [10].

To examine the role of H3.3K27 in *Drosophila* development, we mutated the endogenous *H3*.*3B* gene to generate a non-modifiable K27 residue (*H3*.*3B*^*K27R*^). Females heterozygous for *H3*.*3B*^*K27R*^ exhibit no developmental defects and survive to adulthood with equal frequency as wild type or heterozygous *H3*.*3B*^*null*^ flies (**Fig 1C**). By contrast, males hemizygous for *H3*.*3B*^*K27R*^ demonstrate significantly reduced viability relative to wild type or *H3*.*3B*^*null*^ flies (**Fig 1C**). The few *H3*.*3B*^*K27R*^ males that survive to adulthood are infertile, and longevity assays revealed that they have a markedly shortened lifespan (**Fig 1D**). The infertility of *H3*.*3B*^*K27R*^ males precluded us from generating homozygous *H3*.*3B*^*K27R*^ females. Examination of the lethal phase of *H3*.*3B*^*K27R*^ males revealed that they die almost exclusively during the pupal stage of development (**Fig 1E**). *H3*.*3B*^*K27R*^ males removed from the pupal case just prior to eclosion (96 – 108 hours after pupal formation, hereafter “pharate adults”) exhibit no obvious morphological defects relative to wild type males at the equivalent developmental stage. Males that eclose from their pupal cases are also wild type in appearance, however they exhibit severely restricted movement (not shown). Thus, we conclude that *H3*.*3B*^*K27R*^ causes male-specific lethality near the end of pupal development. The more severe phenotype of *H3*.*3B*^*K27R*^ males relative to *H3*.*3B*^*null*^ males suggests that H3.3BK27R histones are incorporated into chromatin, but that they lack a required functionality provided by wild type H3.3 histones.

### H3.3A and H3.3B are non-equivalent genes

**Fig 2:**
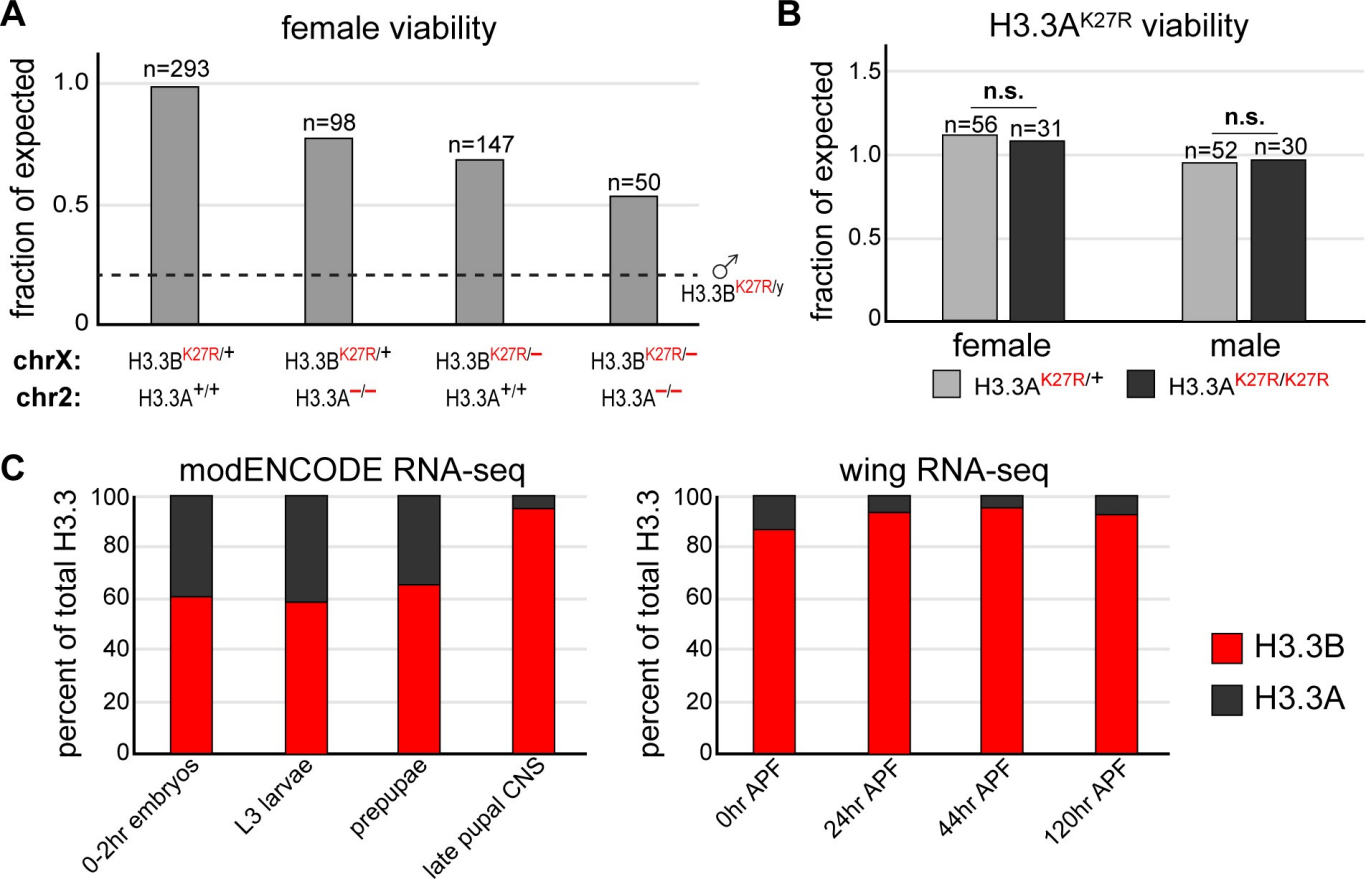
*H3*.*3A* and *H3*.*3B* are non-equivalent genes. (**A**) Bar plot of female viability for the following genotypes: *H3*.*3B*^*K27R*^ heterozygotes, *H3*.*3B*^*K27R*^ heterozygotes also homozygous for an *H3*.*3A* null allele, trans-heterozygotes for *H3*.*3B*^*K27R*^ and *H3*.*3B* null alleles, trans-heterozygotes for *H3*.*3B*^*K27R*^ and *H3*.*3B* null alleles also homozygous null for *H3*.*3A*. The dashed line indicates *H3*.*3B*^*K27R*^ male viability from Fig 1C. n equals the number of adults scored for each indicated genotype. (**B**) Bar plot of viability for males and females heterozygous (light gray) or homozygous (dark gray) for an *H3*.*3A*^*K27R*^ allele. n equals the number of adults scored for each indicated genotype. (**C**) Stacked bar charts of H3.3B and H3.3A RNA-seq signal from selected modENCODE developmental stages and tissues (left) and four time points in wing development (right).

Males and females exhibit different susceptibilities to the *H3*.*3B*^*K27R*^ mutation (**Fig 1C**). Because *H3*.*3B* is located on the X chromosome, females may be less susceptible due to expression of the wild type *H3*.*3B* allele present on their homologous X. Similarly, expression of wild type *H3*.*3A* might suppress *H3*.*3B*^*K27R*^ mutant phenotypes in both males and females. Therefore, we asked if removing additional H3.3 gene copies would increase susceptibility to *H3*.*3B*^*K27R*^ in females by crossing in null alleles of the *H3*.*3A* and *H3*.*3B* genes. Removal of the homologous *H3*.*3B* allele (*H3*.*3B*^*K27R/–*^) decreased *H3*.*3B*^*K27R*^ female viability relative to *H3*.*3B*^*K27R*^ females with a wild type *H3*.*3B* allele (*H3*.*3B*^*K27R/+*^) (**Fig 2A**). Surprisingly, removal of both alleles of *H3*.*3A* from *H3*.*3B*^*K27R*^ females (*H3*.*3B*^*K27R/+*^ *; H3*.*3A*^*–/–*^) reduced viability to a similar extent as removing only one copy of *H3*.*3B*. Finally, removal of all wild type H3.3 copies (*H3*.*3B*^*K27R/–*^*; H3*.*3A*^*–/–*^*)* decreased viability of *H3*.*3B*^*K27R*^ females even further (**Fig 2A**). These findings indicate that *H3*.*3A* and *H3*.*3B* both contribute to suppression of the *H3*.*3B*^*K27R*^ mutant phenotype in females, consistent with the predicted overlapping functions of these genes. However, the magnitude of the contribution made by *H3*.*3B* is greater than that made by *H3*.*3A*.

The relatively minor impact that deletion of *H3*.*3A* had on *H3*.*3B*^*K27R*^ female viability suggests that the *H3*.*3A* and *H3*.*3B* genes are not equivalent. To directly test the role of K27 in H3.3A function, we used CRISPR/Cas9 to generate an *H3*.*3A*^*K27R*^ mutation. Homozygous *H3*.*3A*^*K27R*^ male and female flies are fertile, exhibit no visible defects, and survive to adulthood at similar frequencies as their heterozygous siblings (**Fig 2B**). In fact, *H3*.*3A*^*K27R*^ homozygous flies can be propagated as a stable stock. Because the *H3*.*3A* and *H3*.*3B* genes encode identical proteins, it is unlikely that they perform different biochemical functions *in vivo*. Therefore, we reasoned that differences in *H3*.*3A* and *H3*.*3B* gene function are a consequence of distinct gene expression profiles. Supporting this hypothesis, RNA-seq data from modENCODE reveal that *H3*.*3A* and *H3*.*3B* are expressed at different levels over developmental time, and between different tissues (**Fig 2C**) [40]. This finding is consistent with recent observations from *C*. *elegans*, in which non-allelic H3.3 gene expression patterns range from ubiquitous to germ line-specific [41]. Furthermore, our own RNA-seq data from dissected wings show that steady state *H3*.*3B* RNA levels are significantly greater than those of *H3*.*3A* (**Fig 2C**) [42, 43]. Thus, the more severe phenotype of *H3*.*3B*^*K27R*^ is likely due to higher levels of H3.3B protein relative to H3.3A, as opposed to K27 performing a distinct function in H3.3B relative to H3.3A. Finally, we created a double *H3*.*3*^*K27R*^ mutant genotype by crossing *H3*.*3B*^*K27R*^ and *H3*.*3A*^*K27R*^ flies. Females heterozygous for *H3*.*3B*^*K27R*^ with homozygous *H3*.*3A*^*K27R*^ mutations (*H3*.*3B*^*K27R/+*^ *; H3*.*3A*^*K27R/K27R*^) survive at the expected frequency and are fertile, consistent with a secondary role of *H3*.*3A* relative to *H3*.*3B* (not shown). Notably, we did not recover any *H3*.*3B*^*K27R*^ males with homozygous *H3*.*3A*^*K27R*^ mutations (*H3*.*3B*^*K27R/y*^ *; H3*.*3A*^*K27R/K27R*^), indicating that the presence of wild type H3.3A histones contributes to the survival of *H3*.*3B*^*K27R*^ males.

### Male-specific lethality of H3.3B^K27R^ is a consequence of X-chromosome dosage compensation

**Fig 3:**
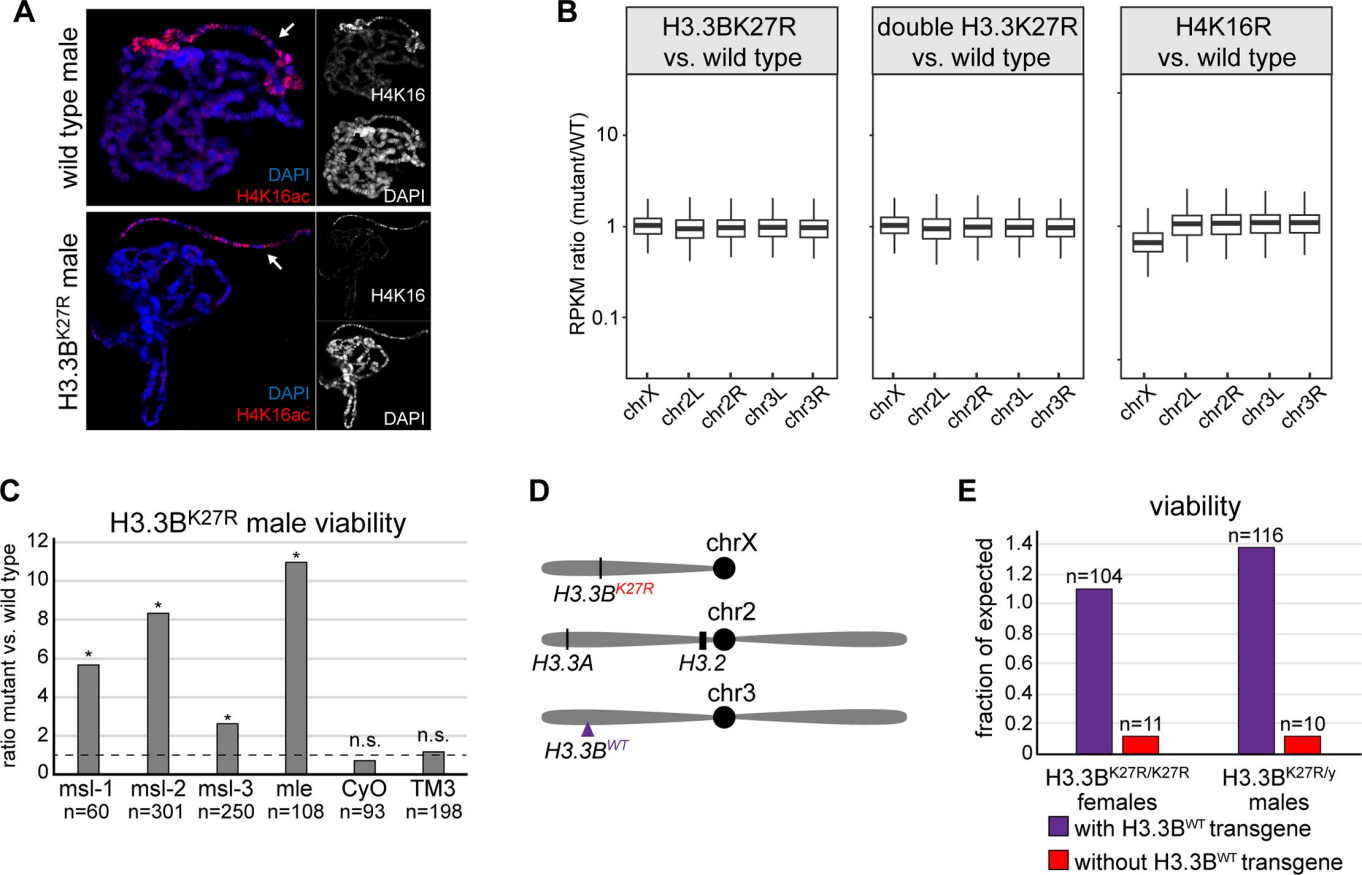
Levels of H3.3BK27R mutant histones determine the impact on development. (**A**) Confocal images of salivary glands from wild type and *H3*.*3B*^*K27R*^ male third instar larvae stained for H4K16ac and DAPI. Arrow indicates the X chromosome. (**B**) Box plots of RNA-seq values for genes located on the X chromosome and the four main autosomal arms. The ratio of RPKM values in mutant versus wild type is plotted for each gene. H4K16 mutant data are from [45]. (**C**) Bar plot of *H3*.*3B*^*K27R*^ male viability for the six indicated genotypes. Data are plotted as the ratio of *H3*.*3B*^*K27R*^ males inheriting the chromosome indicated under each bar relative to its homologous wild type chromosome. Asterisks indicate statistical significance by chi-square test (p values as follows: *msl-1*: 9.6x10^-4^, *msl-2*: 3.2x10^-5^, *msl-3*: 4.4x10^-3^, *mle*: 9.1x10^-4^, *CyO*: 0.37, *TM3*: 0.69). The dashed line indicates the expected ratio of 1. n equals the total number of males scored. (**D**) Cartoon of the genomic location of the *H3*.*3B*^*WT*^ transgene relative to the endogenous *H3* gene loci. (**E**) Bar plot of viability for homozygous *H3*.*3B*^*K27R*^ females and hemizygous *H3*.*3B*^*K27R*^ males inheriting the *H3*.*3B*^*WT*^ transgenic chromosome (purple) or the non-transgenic homologous chromosome (red). n equals the number of adults scored for each indicated genotype.

A key finding from the preceding experiments is that females bearing only one copy of *H3*.*3B*^*K27R*^ and no wild type *H3*.*3* genes (*H3*.*3B*^*K27R/–*^*; H3*.*3A*^*–/–*^*)* survive at a greater frequency than *H3*.*3B*^*K27R*^ males that have two wild type copies of *H3*.*3A* (**Fig 2A, dashed line**). One possible explanation for why males are more sensitive to H3.3BK27R mutant histones than females is X chromosome dosage compensation. In *Drosophila*, transcription of the male X chromosome is hyper-activated approximately two-fold to balance X-linked gene expression between males and females [44]. Therefore, increased lethality of *H3*.*3B*^*K27R*^ males relative to females might be due to a defect in dosage compensation caused by H3.3BK27R mutant histones. Alternatively, increased lethality of *H3*.*3B*^*K27R*^ males could be a consequence of increased expression of *H3*.*3B*^*K27R*^ mutant histone genes due to hyper-activation of X-linked genes in males relative to females. To distinguish between these alternatives, we first performed immunofluorescence on salivary glands from *H3*.*3B*^*K27R*^ males. As part of the dosage compensation pathway, Histone H4 lysine 16 (H4K16) is hyper-acetylated on the male X chromosome relative to autosomes. We observed that H4K16ac levels were indistinguishable between wild type and *H3*.*3B*^*K27R*^ males (**Fig 3A**), indicating that H3.3BK27R mutant histones do not interfere with hyper-acetylation of the male X chromosome. Consistent with this finding, transcriptomic analyses of wild type and *H3*.*3B*^*K27R*^ males (see below) did not reveal a systemic decrease in X-linked gene expression relative to autosomes (**Fig 3B**). RNA-seq values from flies in which the male X chromosome exhibits a dosage compensation defect (mutation of replication-dependent histone H4 lysine 16) are also plotted as a comparison [45]. Together, these data indicate that lethality of *H3*.*3B*^*K27R*^ males is not caused by a defect in dosage compensation.

We next asked if increased lethality of *H3*.*3B*^*K27R*^ males relative to females is a consequence of hyper-activation of the male X chromosome by screening for genetic interactions between *H3*.*3B*^*K27R*^ and dosage compensation mutants. We reasoned that a decrease in dosage compensation might suppress the *H3*.*3B*^*K27R*^ male-specific lethal phenotype by reducing H3.3B expression, as has been previously observed in RNAi experiments for dosage compensation genes [46] (**S1A Fig**). We found that viability was significantly increased for *H3*.*3B*^*K27R*^ males that were also heterozygous for one of several mutations that attenuate dosage compensation (**Fig 3C**). For example, we recovered eleven times more adult *H3*.*3B*^*K27R*^ males that were heterozygous for a mutation in the dosage compensation gene *maleless* (*mle*) (*H3*.*3B*^*K27R/y*^ *; mle*^*9*^*/+*) relative to their siblings with two wild type *mle* genes (*H3*.*3B*^*K27R/y*^ *; CyO/+*). These findings show that the increased sensitivity of males to H3.3BK27R mutant histones relative to females is caused by hyper-activation of the male X chromosome, rather than by a defect in dosage compensation caused by H3.3K27R mutant histones. They also suggest that the relative abundance of H3.3K27R histones determines the impact on development, rather than a male-specific function for H3.3 lysine 27. To further explore potential male-specific functions of H3.3B lysine 27, we introduced a transgene containing the wild type *H3*.*3B* gene onto chromosome three [47] (**Fig 3D**). The additional wild type copy of H3.3B fully rescued viability of H3.3B^K27R^ males (**Fig 3E**). Moreover, these males are now fertile, allowing us to test the consequence of mutating both alleles of H3.3B lysine 27 in females. Homozygous *H3*.*3B*^*K27R*^ females exhibited a similar reduction in viability as *H3*.*3B*^*K27R*^ males, supporting the conclusion that H3.3B lysine 27 does not have a male-specific function. Instead, we conclude that levels of H3.3BK27R mutant histones above a certain threshold impairs development in both males and females.

### H3.3B^K27R^ males exhibit diminished PTM levels

To determine the consequences of *H3*.*3B*^*K27R*^ mutation at the molecular level, we performed western blotting for PTMs associated with H3 lysine 27. H3K27 can be both acetylated and methylated. H3K27ac is correlated with transcriptional activation [13], whereas H3K27me3 and H3K27me2 are associated with transcriptional silencing [16, 48]. The amino acid sequence surrounding lysine 27 is identical in both H3.2 and H3.3, suggesting that the enzymes that catalyze these PTMs would be capable of recognizing either histone as a substrate. Supporting this expectation, mass spectrometry results from *Drosophila* and mammalian cell lines indicate that both H3.2 and H3.3 can be acetylated or methylated on lysine 27 [30, 31]. However, the abundance of these PTMs on H3.2 and H3.3 is not equivalent. The majority of acetylation is found on H3.3, consistent with the relative enrichment of H3.3 at transcriptionally-active loci [27]. By contrast, the majority of di- and tri-methylation is found on H3.2, consistent with the relative depletion of H3.3 in transcriptionally-silenced loci [30, 31].

**Fig 4:**
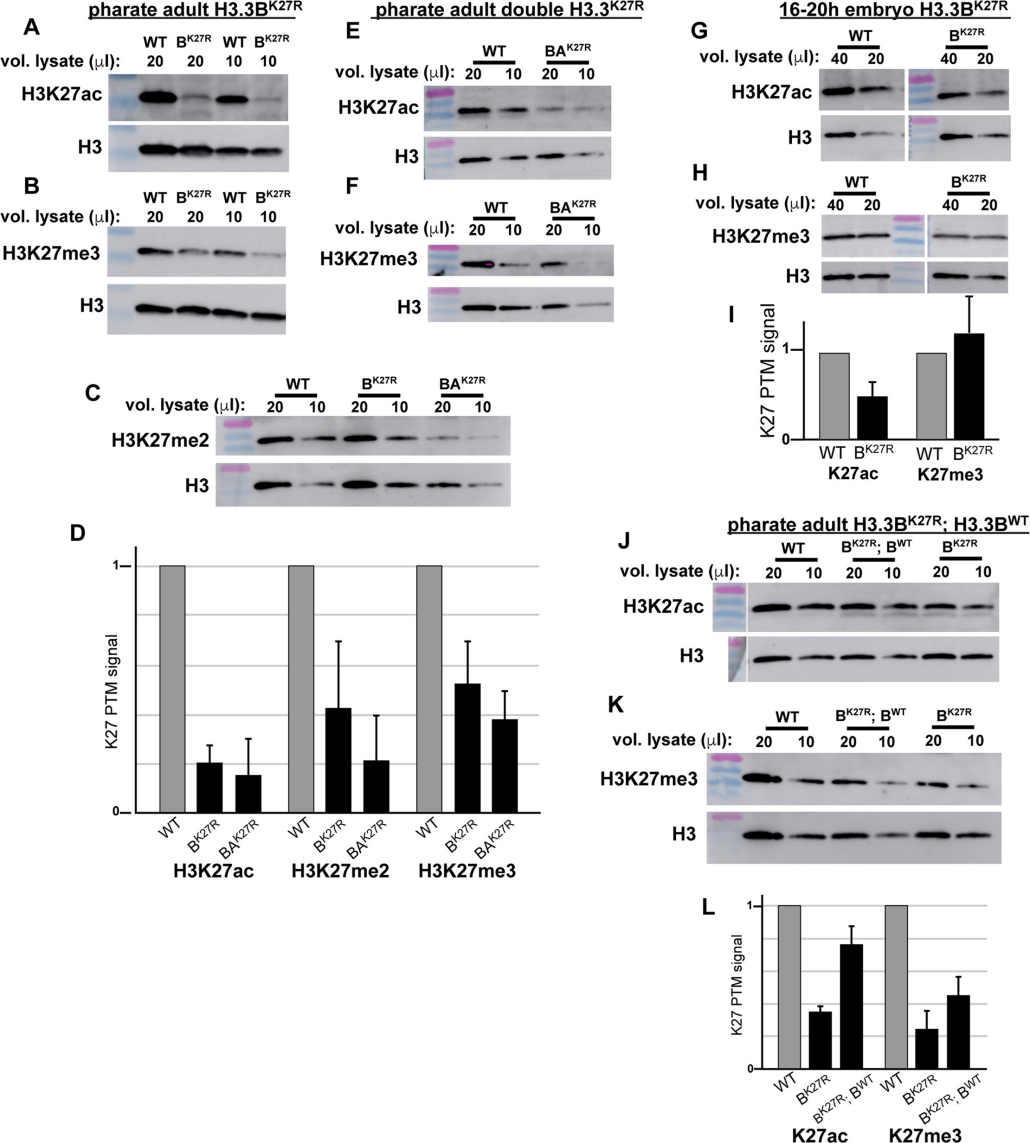
Histone modifications are diminished in *H3*.*3*^*K27R*^ flies. Western blots of H3K27ac (**A, E, G, J**), H3K27me2 (**C**), and H3K27me3 (**B, F, H, K**) from *H3*.*3B*^*K27R*^ (**A**-**C**) and double *H3*.*3*^*K27R*^ **(C, E, F**) pharate adult males, 16–20 hour *H3*.*3B*^*K27R*^ male embryos (**G, H**), and *H3*.*3B*^*K27R*^ pharate adult males rescued by the *H3*.*3B*^*WT*^ transgene (**J, K**). (**D, I, L**) Bar plots depicting the average fold change in H3K27 PTM signal relative to wild type (WT). Each lane was also normalized to total H3 signal. Error bars represent standard deviation of at least two independent western blots.

To determine if *H3*.*3*^*K27R*^ males exhibit any changes in H3K27 PTM levels, we performed western blotting on pharate adult males, just prior to the time when *H3*.*3B*^*K27R*^ males die. *H3*.*3B*^*K27R*^ pharate adult males display a 4.8-fold decrease in H3K27ac levels relative to wild type males (**Fig 4A and 4D**). *H3*.*3B*^*K27R*^ pharate adult males also display a 1.9-fold decrease in H3K27me3 levels (**Fig 4B and 4D**), and a 2.3-fold decrease in H3K27me2 levels (**Fig 4C and 4D**). Total levels of H3 were unaffected, further supporting that H3.3BK27R mutant histones are incorporated into chromatin but do not support K27 modification. Thus, even though prior work from *Drosophila* cell lines indicates that H3.3 accounts for the minority of total histone H3 levels [30], H3.3K27 is required for a significant fraction of the total H3K27ac, H3K27me2 and H3K27me3 levels, at least in pharate adults. Moreover, these data indicate that neither H3.2K27 nor H3.3AK27 fully substitutes for the absence of H3.3BK27 in these flies. Western blot analysis of double *H3*.*3*^*K27R*^ pharate adult males revealed further decreases in K27 PTM levels (**Fig 4C–4F**). Double *H3*.*3*^*K27R*^ males display a 6.4-fold decrease in H3K27ac levels (**Fig 4D and 4E**), a 4.7-fold decrease in H3K27me2 levels (**Fig 4C and 4D**), and a 2.6-fold decrease in H3K27me3 levels (**Fig 4D and 4F**). Because H3.3 is expressed in a replication-independent manner, whereas H3.2 expression is coupled with DNA replication, we reasoned that H3.3 may account for a greater fraction of the total H3 levels at later stages of development, such as in pharate adults, when many cells have transitioned into a post-mitotic state. To test this hypothesis, we examined an earlier stage of development when most cells are still replicating. Western blot analysis of 16–20 hour embryos revealed no significant difference in H3K27me3 levels and a modest decrease in H3K27ac levels in *H3*.*3B*^*K27R*^ relative to wild type genotypes **(Fig 4G–4I).** Thus, the defects in H3K27 PTM levels exhibited by *H3*.*3B*^*K27R*^ males are more apparent at later stages of development, consistent with an increased fraction of H3K27 PTMs occurring on H3.3 over time. Finally, we performed western blots on *H3*.*3B*^*K27R*^ males rescued by the *H3*.*3B*^*WT*^ transgene and observed increases in both H3K27me3 and H3K27ac levels (**Fig 4J–4L**). Restoration of H3K27 PTMs by expression of the *H3*.*3B*^*WT*^ transgene strongly argues that lethality of *H3*.*3B*^*K27R*^ males is determined by the level of H3.3K27R mutant histones incorporated into chromatin and the concomitant loss of any functions mediated by H3.3K27 modification.

### RNA-seq reveals no global gene expression changes in H3.3^K27R^ males

**Fig 5:**
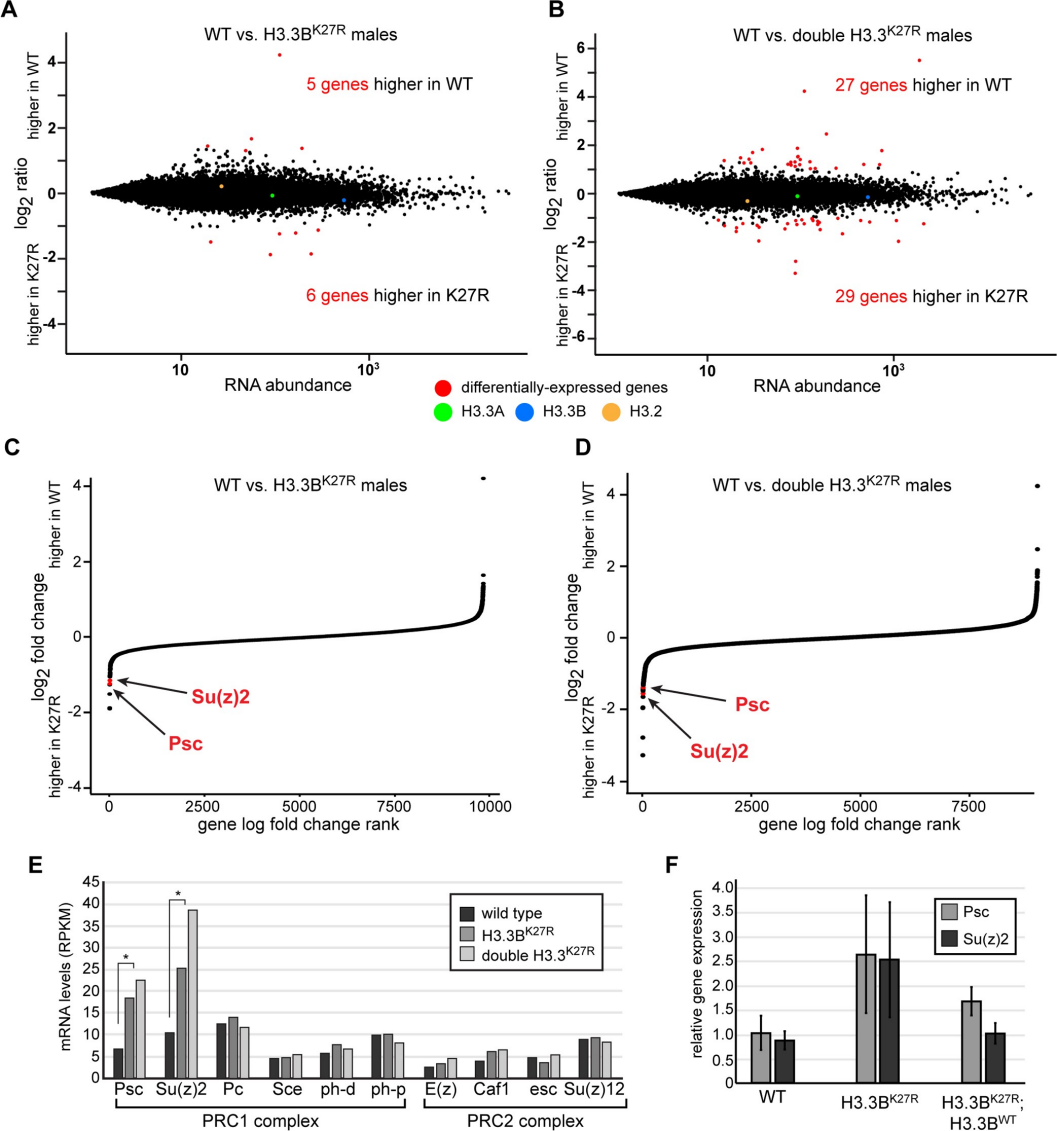
Polycomb target genes are de-repressed in *H3*.*3*^*K27R*^ flies. (**A**, **B**) MA plots of RNA-seq signal in annotated genes for wild type (WT) relative to *H3*.*3B*^*K27R*^ males (**A**) and double *H3*.*3*^*K27R*^ males (**B**). Differentially-expressed genes are indicated in red. RNA-seq signal for each *H3* gene is also indicated. (**C**, **D**) Plots of the log-fold change in RNA-seq signal relative to wild type for genes in *H3*.*3B*^*K27R*^ males (**C**) and double *H3*.*3*^*K27R*^ males (**D**), ranked by log-fold change. Genes for which an adjusted p value could not be calculated were excluded. *Psc* and *Su(z)2* are indicated in red. (**E**) Bar plot of RPKM values for core members of the PRC1 and PRC2 Polycomb complexes in wild type, *H3*.*3B*^*K27R*^, and double *H3*.*3*^*K27R*^ males. Asterisks indicate *Psc* and *Su(z)2* were identified as differentially-expressed relative to wild type by DEseq2 (see S1 and S2 Tables for p values). (**F**) Bar plot of real-time RT-PCR results from the three indicated genotypes. Error bars represent the standard deviation of two biological replicates, performed in triplicate.

The above experiments revealed a significant decrease in H3K27ac and H3K27me2/3 in *H3*.*3B*^*K27R*^ males at the phenocritical stage. To determine whether these PTM changes are associated with changes in gene expression, we performed RNA-seq on whole pharate adult males. Inspection of the reads mapping to the H3.3 genes confirmed the genotypes of our CRISPR mutants (**S1B Fig**). Surprisingly, comparisons between wild type and *H3*.*3B*^*K27R*^ males revealed relatively few changes in gene expression (**Fig 5**). Only five genes were decreased in *H3*.*3B*^*K27R*^ males relative to wild type, and only six genes were increased in *H3*.*3B*^*K27R*^ males (**Fig 5A**). Mutation of both H3.3 genes resulted in only 27 genes with decreased expression and 29 genes with increased expression in double *H3*.*3*^*K27R*^ males (**Fig 5B**). Perhaps more important than the differences observed between wild type and *H3*.*3*^*K27R*^ males were the differences that were not observed. Despite complete loss of H3.3K27ac, and a nearly 4-fold decrease in total H3K27ac levels, we did not detect any global changes in steady-state RNA levels. Considering that H3K27ac levels are positively correlated with gene expression levels [49] [13], highly-expressed genes may be particularly sensitive to loss of H3K27ac. However, we did not observe any relative changes in gene expression between highly- and lowly-expressed genes in *H3*.*3*^*K27R*^ males. This finding was supported by the inclusion of standardized RNA spike-in controls, which revealed no global differences in dynamic range between wild type and *H3*.*3*^*K27R*^ RNA-seq signal (**S1C Fig**). The lack of global changes in steady-state RNA levels in *H3*.*3*^*K27R*^ flies strongly contrasts with our previous observations from *H3*^*K36R*^ flies, which exhibited a systematic decrease in highly-expressed genes and a systematic increase in lowly-expressed genes relative to wild type flies [50]. We conclude that loss of H3.3K27ac does not have a global impact on gene expression. Because H3.3 accumulates to high levels at regulatory loci, and it is enriched for active PTMs such as K27ac, this finding suggests that H3.3K27ac is not essential for proper enhancer or promoter function. Notably, levels of the replication-coupled histone H3.2 gene were unchanged in *H3*.*3*^*K27R*^ males relative to wild type (**Fig 5A and 5B**), indicating that H3.2 does not compensate for *H3*.*3*^*K27*^ mutation, at least at the level of RNA, unlike the situation in H3.3 null flies, which exhibited increased levels of H3.2 RNA [39].

### Polycomb targets are among the few differentially-expressed genes in H3.3^K27R^ males

Despite a lack of global changes in gene expression, we nevertheless sought to determine if any of the differentially-expressed genes in *H3*.*3*^*K27R*^ males could help to explain the mutant phenotype. In particular, we looked for genes that were differentially expressed in both *H3*.*3B*^*K27R*^ and double *H3*.*3*^*K27R*^ mutant males relative to wild type, reasoning that these would be most relevant for understanding H3.3K27 function. Nearly two-thirds (7 out of 11) of genes differentially expressed in *H3*.*3B*^*K27R*^ males are also differentially expressed in *H3*.*3*^*K27R*^ double mutants (**S1 Table, S2 Table**). Among these are two Polycomb target genes, *Psc* and *Su(z)2*, that are more highly expressed in K27R mutants than in wild type (**Fig 5C and 5D**). Notably, the levels of *Psc* and *Su(z)2* are increased further in *H3*.*3*^*K27R*^ double mutants relative to *H3*.*3B*^*K27R*^ males (**Fig 5E**). *Psc* and *Su(z)2* encode genes that share homology and function. Both genes are bound by Polycomb group proteins and display high levels of H3K27me3 *in vivo* [51, 52]. In Polycomb group mutants, expression of both *Psc* and *Su(z)2* increase, indicating that these genes are repressed by Polycomb group activity [53]. Interestingly, Psc is itself a core component of the PRC1 complex [54] [55], and it functions in Polycomb group target gene repression at least in part by compacting chromatin [56]. The role of Psc in PRC1 activity can be compensated for by Su(z)2 [57]. Thus, *Psc* and *Su(z)2* have been proposed to be part of a feedback mechanism that adjusts the levels of PRC1 activity [52, 53]. According to this model, if Polycomb group function decreases below a threshold, transcription of *Psc* and *Su(z)2* increases, thereby augmenting PRC1 activity. *Psc* and *Su(z)2* are the only members of the PRC1 or PRC2 complexes that are differentially expressed in *H3*.*3*^*K27R*^ males (**Fig 5E**). To determine if de-repression of *Psc* and *Su(z)2* is suppressed in *H3*.*3B*^*K27R*^ males rescued by the *H3*.*3B*^*WT*^ transgene, we performed quantitative RT-PCR on RNA collected from pharate males. Expression of *Su(z)2*, and to a lesser extent *Psc*, is fully restored by the *H3*.*3B*^*WT*^ transgene (**Fig 5F**), further supporting the conclusion that levels of H3.3K27R mutant histones beyond a certain threshold impair development. These findings also demonstrate that increased expression of *Psc* and *Su(z)2* is not due to off-target effects caused by CRISPR mutagenesis. Together, these results indicate that Polycomb group activity is compromised in *H3*.*3B*^*K27R*^ males, and that mutation of all copies of H3.3K27 exacerbates this effect, suggesting that H3.3K27 is required for proper Polycomb group function.

### H3.3B^K27R^ enhances Polycomb mutant phenotypes

**Fig 6:**
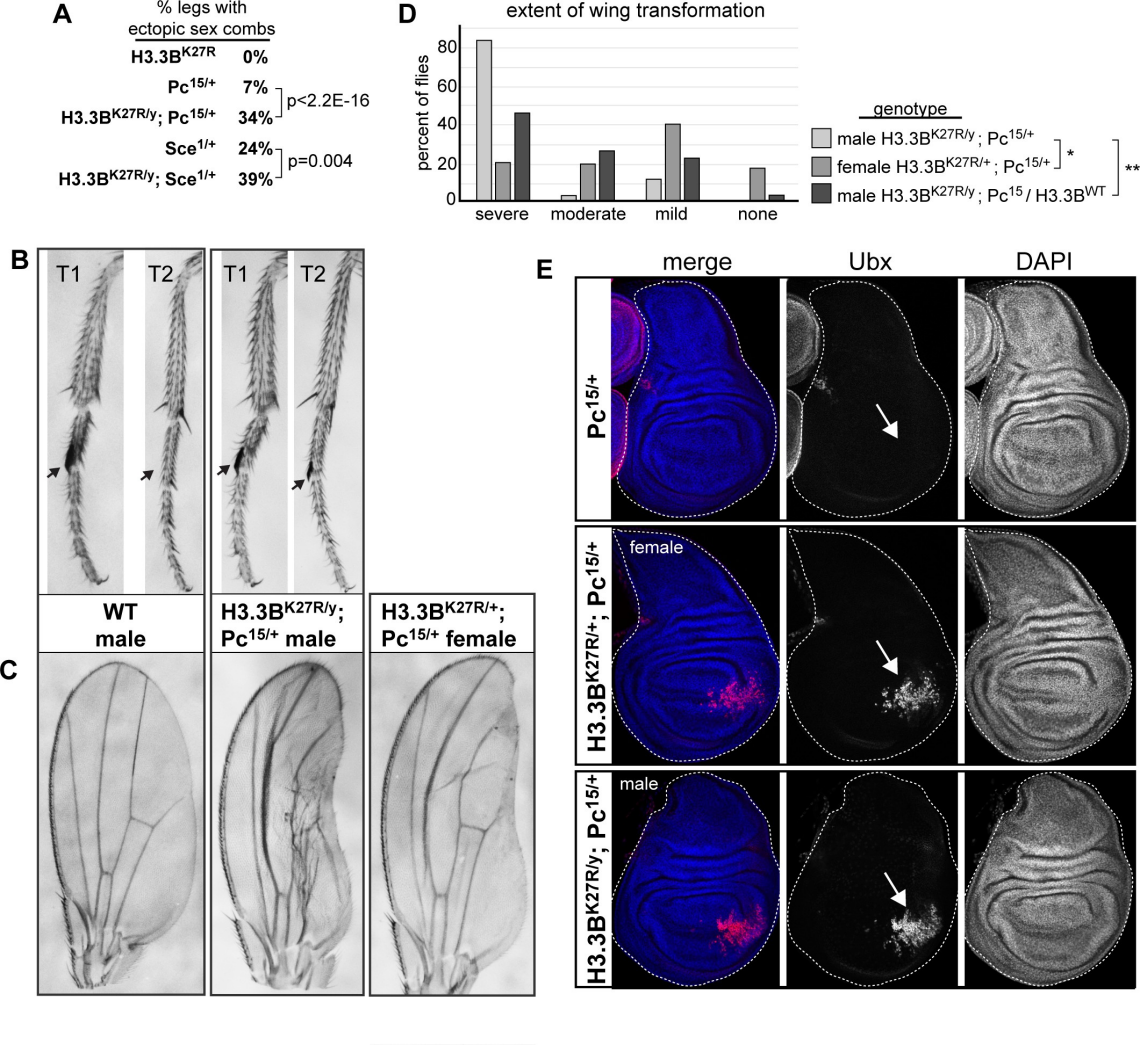
*H3*.*3B*^*K27R*^ enhances *Polycomb* mutant phenotypes. (**A**) Table quantifying the frequency of ectopic sex combs for the four indicated genotypes. P values were obtained from a Fisher’s Exact Test. (**B**) Bright field images of first and second thoracic legs from wild type and *H3*.*3B*^*K27R*^ hemizygous males that are also heterozygous for a *Polycomb* null allele. Arrows indicate the location where sex combs develop. (**C**) Bright field images of adult wings from wild type, *H3*.*3B*^*K27R*^ hemizygous males also heterozygous for a *Polycomb* null allele, and *H3*.*3B*^*K27R*^ heterozygous females also heterozygous for a *Polycomb* null allele. (**D**) Bar plots quantifying the severity of wing defects from the three indicated genotypes. * indicates p value of 2.7E-3, ** indicates p value 1.2E-6 (ordinal logistic regression). (**E**) Confocal images of wing imaginal discs stained for DAPI (blue) and Ubx (red) for the indicated genotypes. Arrows indicate the location of ectopic Ubx expression.

The above RNA-seq analysis revealed increased expression of *Psc* and *Su(z)2*, two Polycomb group target genes, suggesting a defect in Polycomb group pathway activity in *H3*.*3*^*K27R*^ mutants. To further explore the potential role of H3.3K27 in Polycomb group target gene regulation, we looked for evidence of genetic interactions between *H3*.*3B*^*K27R*^ and mutants in components of the Polycomb or Trithorax group complexes (**Table 1**). We reasoned that a reduction in Polycomb activity might enhance the *H3*.*3B*^*K27R*^ mutant phenotype, whereas reduced activity of Trithorax, which functions to promote activation of many Polycomb target genes, might suppress the *H3*.*3B*^*K27R*^ mutant phenotype. This type of genetic interaction approach has been extensively used to identify new Polycomb and Trithorax group genes. Although we did not observe any genetic interactions between Trithorax group genes and *H3*.*3B*^*K27R*^, we identified two Polycomb group components, *Polycomb* (*Pc*) and *Sex combs extra* (*Sce*), whose mutation enhanced the *H3*.*3B*^*K27R*^ mutant phenotype. Both *Pc* and *Sce* encode core components of the PRC1 complex. The chromodomain of Pc specifically binds H3K27me3 histones [20, 21], whereas Sce contains a RING finger domain that mono-ubiquitylates histone H2A, a mark closely correlated with Polycomb target gene repression [8, 58]. We found that *H3*.*3B*^*K27R*^ males that were also heterozygous for an *Sce* null mutation displayed an increased frequency of ectopic sex combs (**Fig 6A**). *H3*.*3B*^*K27R*^ males heterozygous for a *Pc* null mutation exhibited several classic Polycomb phenotypes, including ectopic sex combs (**Fig 6B**) and defects in posterior wing morphology (**Fig 6C**). Neither of these phenotypes were observed in otherwise wild type *H3*.*3B*^*K27R*^ males. Remarkably, we also observed defects in posterior wing morphology in females heterozygous for both *Pc* and *H3*.*3B*^*K27R*^ (**Fig 6A**), however the severity of wing defects in females was diminished relative to males (**Fig 6D**), consistent with a dose-dependent impact of H3.3BK27R histones on mutant phenotypes. Similarly, the severity of wing defects in males was partially suppressed by expression of H3.3B^WT^ from the rescue transgene (**Fig 6D**). Posterior wing defects in Polycomb group mutants are interpreted as partial transformation toward haltere identity due to failure to maintain silencing of the Polycomb group target gene *Ubx* in the wing. Supporting this conclusion, immunostaining of third instar wing imaginal discs revealed Ubx expression in the posterior compartment of *H3*.*3B*^*K27R*^ males and females that were also heterozygous for *Pc* (**Fig 6D**). Thus, the presence of H3.3B^K27R^ mutant histones enhances both *Sce* and *Pc* mutant phenotypes. Since *Sce* and *Pc* both encode core components of the PRC1 complex, which specifically binds H3K27me3 histones, these findings suggest that the histone PTM reader of the Polycomb pathway is particularly sensitive to H3.3K27R mutant histones. We conclude that H3.3BK27 contributes to silencing of Polycomb group target genes during normal development.

**Table 1:** List of genes tested for genetic interactions with *H3*.*3B*^*K27R*^. Specific alleles used are listed in the Materials and Methods. “–”indicates no genetic interaction observed.

gene name	function	genetic interaction with *H3*.*3B*^*K27R*^
***Pc***	Polycomb, PRC1, H3K27me3 binding	Enhancement
***Sce***	Polycomb, PRC1, H2AK119 ubiquitination	Enhancement
***Psc***	Polycomb, PRC1, chromatin compaction	–
***Su(z)2***	Polycomb, PRC1, chromatin compaction	–
***E(z)***	Polycomb, PRC2, H3K27 methyltransferase	–
***esc***	Polycomb, PRC2, H3K27me3 binding	–
***Su(z)12***	Polycomb, PRC2, nucleosome binding	–
***brm***	Brahma Associated Proteins Complex, ATPase	–
***trx***	Trithorax, TAC1 Complexes, H3K4 methyltransferase	–
***Utx***	Trithorax Related Complex, H3K27 demethylase	–
***ash2***	Trithorax, Trithorax Related, Compass Complexes, H3K4 methyltransferase	–

## Discussion

Post-translational modification of H3K27 is strongly correlated with both active transcription (H3K27ac) and epigenetic silencing (H3K27me3). The histones bearing these PTMs are encoded by both replication-dependent and replication-independent histone genes. H3K27ac is enriched on replication-independent H3.3 due to the high degree of nucleosome turnover in active chromatin. Conversely, H3K27me3 is enriched on replication-dependent histones due to the low rate of nucleosome turnover across Polycomb target genes. Despite these correlations, our data demonstrate that mutation of H3.3K27 does not impair global gene expression profiles, indicating that H3.3K27 modification is dispensable for transcriptional activation. Instead, this work reveals that H3.3K27 contributes to epigenetic silencing by the Polycomb group proteins.

### Genetic interrogation of histone residue function

Much of our knowledge of histone PTM function in animals has come from interpreting the mutant phenotypes of proteins that catalyze or bind specific PTMs. However, mutations in these writer and reader proteins may not reveal the function of a given histone PTM. Many histone-modifying enzymes have multiple substrates [59], meaning that mutation of these enzymes will result in loss of PTMs on all targets, in addition to histones. For example, E(z) and its mammalian homologues EZH1/2 methylate multiple proteins in addition to histone H3, including gene regulatory proteins such as transcription factors [60] [61], elongation factors [62], and other chromatin-binding proteins [63]. The E(z) mutant phenotype is thus a consequence of the loss of methylation on each of its substrates, not only the loss of methylation on H3K27. Likewise, mutations in histone PTM binding proteins can also cause indirect effects. Many histone PTM readers (and writers) belong to multiprotein complexes that become destabilized in the absence of a component. For example, mutations of the PRC1 component Sce exhibit global decreases in the levels of Polycomb, another PRC1 component that is required for complex activity [8]. Similar findings have been reported for H3K4-specific methyltransferases in *Drosophila* and mammalian systems [64, 65]. For these reasons, a more direct method of determining histone PTM function is to introduce mutations that prevent residue modification in the histones themselves.

Here, we have used CRISPR/Cas9 to mutate K27 of the replication-independent histone H3.3, allowing us to directly test its role in normal development. Previous genetic characterization of H3.3 has utilized either protein null mutants [38, 39], or loss-of-function mutations in the chaperones that facilitate H3.3 deposition into chromatin [66, 67]. These efforts have revealed important roles for H3.3 in paternal chromatin assembly during fertilization [68], chromatin transitions in the male germline [39], and for proper boundary element function [69]. However, failure to incorporate H3.3 into chromatin may mask some of its functions due to compensatory effects by the replication-dependent histone genes. For example, the levels of H3.2 gene expression are elevated in H3.3 null flies [39]. Because H3.2 can functionally replace H3.3 [10], previous studies may have under-estimated the role of H3.3 in development and homeostasis. More recently, the discovery of H3.3K27 missense mutations in pediatric glioma patients has led to analysis of methionine substitutions in model systems [36, 70]. However, the role of H3.3K27 in normal development is difficult to discern from these mutations because they are thought to dominantly interfere with PRC2 activity by sequestering or inactivating the methyltransferase [35] [71]. The arginine substitution we have employed here was previously shown not to act by dominantly interfering with methyltransferase activity [35]. Lewis and co-authors demonstrated that transgenic expression of H3.3K27R mutant histones did not result in global loss of H3K27me3 levels upon transgenic expression of H3.3K27M mutant histones. Thus, the H3.3K27R mutant phenotypes we describe here are mechanistically distinct from H3.3K27M mutant phenotypes, and likely reflect the normal role of H3.3K27 in development. Finally, it is unlikely that H3.2 compensates for loss of H3.3K27 because we do not detect an increase in H3.2 steady-state RNA levels in *H3*.*3*^*K27R*^ mutants. Taken together, we conclude that our approach has revealed a previously-unappreciated requirement for H3.3K27 in development.

### Dose-dependent effects of H3.3K27R expression

Our data suggest that increasing concentrations of H3.3K27R histones results in increasingly severe mutant phenotypes. Progressively fewer adults were recovered as additional *H3*.*3* gene copies were removed from *H3*.*3*^*K27R*^ heterozygous females (**Fig 2A**). Thus, as the amount of wild type H3.3 histones decreased relative to H3.3K27R mutant histones, the phenotype became progressively more severe. This suggests that the ratio of mutant to wild type H3.3 is important. Consistent with this interpretation, expression of wild type H3.3B histones from a transgene suppressed the lethality and fertility defects of *H3*.*3B*^*K27R*^ males (**Fig 3E**). However, *H3*.*3B*^*K27R*^ males exhibit a stronger mutant phenotype than females with one *H3*.*3B*^*K27R*^ mutant allele and no wild type *H3*.*3* alleles (**Fig 2A**). Since the male *H3*.*3B* allele is expressed at higher levels than a single female *H3*.*3B* allele (due to dosage compensation), this finding suggests that the amount of H3.3K27R mutant histones relative to the total H3 levels is also important for development.

The dose-dependent sensitivity of flies to H3.3K27R mutant histones may help to explain their late-stage lethal phase. *H3*.*3B*^*K27R*^ males die almost exclusively as late pharate adults (**Fig 1E**). This may be due to a greater reliance on H3.3 in late-stage animals. As development comes to an end, many cells cease dividing, leading to a significant decrease in expression of the replication-dependent histone genes, which are only expressed at high levels during S phase. By contrast, levels of replication-independent histone genes like H3.3 increase in post-mitotic cells, resulting in H3.3 comprising a greater proportion of total histone H3 levels [25, 26]. Thus, as *H3*.*3*^*K27R*^ mutant flies age, it is possible that mutant histone expression surpasses a threshold concentration that results in deregulation of gene expression. This interpretation is consistent with our observation that *H3*.*3*^*K27R*^ mutant males appear morphologically normal, indicating that the patterning and cell fate specification that occurs earlier in development are not affected in *H3*.*3*^*K27R*^ mutant flies. It is also consistent with the greater decrease in H3K27 PTM levels observed in pharate adults relative to the decrease observed in embryos (**Fig 4**). Because we detect differential expression of relatively few genes in whole-animal RNA-seq profiling of *H3*.*3*^*K27R*^ flies, it is possible that gene deregulation occurs only in a subset of cells. However, since the mutation is lethal, proper function of these cells must be essential for viability. Future work will be required to determine which cells are first to manifest the *H3*.*3*^*K27R*^ mutant phenotype. It is possible that neurons or muscles are particularly sensitive since the *H3*.*3*^*K27R*^ mutant flies that succeed in eclosing exhibit reduced locomotion. Neurons are particularly good candidates because many neurons present in the adult become post-mitotic during larval stages, days before the H3.3K27R phenocritical stage, meaning that H3.3 levels are expected to be higher in these cells.

### What role could H3.3K27 play in Polycomb target gene silencing?

Although we show that H3.3K27 is required for proper silencing by Polycomb group proteins, the molecular role played by H3.3K27 in target gene repression remains unknown. Gene loci silenced by Polycomb typically exhibit broad domains of H3K27me3. These domains display low rates of histone replacement [28, 72, 73], resulting in the majority of H3K27me3 being carried by replication-independent H3.2 histones [30, 31]. However, as cells shut down replication-dependent H3.2 gene expression in post-mitotic cells, it is possible that H3.3 is incorporated into H3K27me3 domains, and as these cells age, an increasing proportion of H3K27me3 is found on H3.3. In *H3*.*3*^*K27R*^ mutants, an increased reliance on H3.3 would result in the accumulation of non-modifiable histones and decreasing levels of H3K27me3. Because silencing by Polycomb depends on H3K27me3 dosage, H3.3K27R levels would eventually surpass a threshold in these mutant flies, resulting in de-repression of Polycomb target genes. Our data provide some support for this model, in that we observe a significant decrease in global H3K27me3 levels in pharate adult *H3*.*3*^*K27R*^ mutants, suggesting that wild type H3.3 carries a substantial fraction of H3K27me3 at this late stage of development.

In an alternative model, it is possible that the role H3.3K27 plays in Polycomb target gene silencing resides at *cis*-elements called Polycomb Response Elements (PREs) [74]. PREs recruit Polycomb group complexes to target loci. In contrast to H3K27me3 domains, the chromatin adjacent to PREs is dynamic and exhibits high levels of histone replacement [27]. As a result, nucleosomes flanking PREs are enriched for H3.3. It is possible that modification of these flanking H3.3 histones is important for recruitment of Polycomb group proteins to target loci or for their spread beyond the PRE. In this manner, defects in Polycomb group protein function at PREs caused by mutant H3.3K27R histones could lead to loss of H3K27 methylation across Polycomb target gene loci, thus explaining the global reduction in H3K27me3 and H3K27me2 observed. H3.3K27 could also contribute to histone turnover at PREs. In mouse embryonic stem (ES] cells, H3.3 is required to maintain the dynamic chromatin state found at H3K27me3-marked bivalent promoters [75]. However, this requirement was not dependent on H3.3K27, indicating that the potential role of H3.3K27 at *Drosophila* PREs is mechanistically distinct from H3.3’s role in ES cells.

## Materials and methods

### CRISPR-Cas9 mutagenesis

Mutagenesis was performed essentially as described previously [47]. Briefly, the H3.3B lysine 27 to arginine substitution was made using a single gRNA (TGCCCGTAAGTCGACCGGAGGAAAGG) inserted into *pCFD3*. This construct was co-injected with a 1.9kb repair template containing the *H3*.*3B*^*K27R*^ substitution into strain *yw; attP40{nos-Cas9}/CyO* at BestGene, Inc (Chino Hills, CA). *H3*.*3B*^*K27R*^ alleles were identified using PCR of genomic DNA followed by Hpy99I digestion (H3.3BFor -GTCCACGACACAGCACAACG, H3.3B Rev -CACAGTTCCGGGACGATAAC). In positive strains, the entire 1.9kb region was subjected to Sanger sequencing to verify the absence of additional mutations. Six independent *H3*.*3B*^*K27R*^ mutant lines were isolated. The H3.3A lysine 27 to arginine substation was made using a single gRNA (CAAGGCGCCCCGCAAGCAGCTGG) inserted into *pCFD3*. This construct was co-injected with a 2 kb repair template containing the *H3*.*3A*^*K27R*^ substitution into strain *y*, *M{w[+mC] = nos-Cas9*.*P}ZH-2A w*. *H3*.*3A*^*K27R*^ alleles were identified using PCR of genomic DNA (H3.3A For-GTCGTAAGGGCAAATTCCGTACTC, H3.3A Rev GCGACGAATCTCACGCAGG) followed by Hpy99I digestion and then the entire 2kb region was sequenced. Four independent *H3*.*3A*^*K27R*^ mutant lines were isolated.

### Drosophila genetics

For the viability experiments presented in **Fig 1C**, female *yw/FM7* or *H3*.*3B*^*K27R*^*/FM7-GFP* or *H3*.*3B*^*0*^*/ FM7-GFP* were crossed to male *yw*, and the number of eclosed flies was counted. Data were plotted as a fraction of the total number of eclosed flies for each indicated sex and genotype, divided by the expected fraction. Greater than 1,500 progeny were counted for each cross. For the longevity experiments presented in **Fig 1D**, female *yw/FM7* or *H3*.*3B*^*K27R*^*/FM7-GFP* or *H3*.*3B*^*0*^*/ FM7-GFP* were crossed to male *yw*, and female *H3*.*3B*^*K27R*^*/ FM7-GFP* were crossed to male *H3*.*3B*^*0*^. Newly eclosed adults of the genotype of interest (maximum of 30 flies/vial) were moved to fresh vials daily, and were allowed to mate for 2 to 3 days, after which the mates were removed from the vials. The number of surviving adults was recorded for up to two months. Flies were maintained at 25C and flipped onto fresh food every 3 to 4 days. Numbers represent the total number of adult flies from three independent experiments.

To determine the lethal phase of male *H3*.*3B*^*K27R*^ flies (**Fig 1E**), females of the genotype *yw/ FM7-GFP* or *H3*.*3B*^*K27R*^*/ FM7-GFP* or *H3*.*3B*^*0*^*/ FM7-GFP* were crossed to *FM7-GFP* males. Embryos were collected on apple plates over a 4-hour window and allowed to age at 25C for 16 hours. The total number of embryos was recorded, GFP-negative embryos were counted and transferred to a fresh apple plate. GFP-negative larvae that hatched were counted and transferred to fresh vials. The number of subsequent pupae and eclosed adults was recorded.

To examine the effect of H3.3 gene copy number on *H3*.*3B*^*K27R*^ female viability (**Fig 2A**), the following 4 crosses were performed:

1. *yw / FM7-GFP; Df(2L)BSC110 / CyO x yw; H3*.*3A*^*2x1*^ */ CyO*,
2. *H3*.*3B*^*0*^ */ FM7-GFP; Df(2L)BSC110 / CyO x yw; H3*.*3A*^*2x1*^ */ CyO*
3. *H3*.*3B*^*K27R*^*/ FM7-GFP; Df(2L)BSC110 / CyO x yw; H3*.*3A*^*2x1*^ */ CyO*
4. *H3*.*3B*^*K27R*^*/ FM7-GFP; Df(2L)BSC110 / CyO x H3*.*3B*^*0*^ *; H3*.*3A*^*2x1*^ */ CyO*

Vials were maintained at 25C and flipped every other day. Data were plotted as observed versus expected genotypic frequency based on Mendelian ratios. The genotype of a subset of *H3*.*3B*^*K27R/–*^*; H3*.*3A*^*–/–*^adults was confirmed by PCR. *H3*.*3B*^*0*^ was identified with for- TAAGCATCTAGAATTTTCCTCTTGCCTGCACA and rev- GCTGCCTCCGCGAATTA primers, *H3*.*3A*^*2x1*^ was identified with for- GGGTCACACTGAGCAGACG and rev- GATGTCCTTGGGCATAATGG primers and *Df(2L)BSC110* was identified with for- GAACGAAGCTGATGTGCTATTG and rev- GACATCCGAGTCTTTGCATACT primers.

To determine the effect of *H3*.*3A*^*K27R*^ mutation on viability (**Fig 2B**), females of the genotype *yw/yw; H3*.*3A*^*K27R*^ */ CyO* were crossed to males of the genotype *yw; H3*.*3A*^*K27R*^ */ CyO*. Vials were maintained at 25C and flipped every other day. The number of heterozygous and homozygous *H3*.*3A*^*K27R*^ offspring was recorded.

To test for genetic interactions between *H3*.*3B*^*K27R*^ and dosage compensation mutations (**Fig 3C**), females of the genotype *H3*.*3B*^*K27R*^*/FM7-GFP* were crossed to males of the following genotypes:

1. *msl-1*^*γ216*^, *cn*, *bw / CyO* (Bloomington stock number 5870)
2. *msl-2*^*227*^ */ CyO* (Bloomington stock number 5871)
3. *msl-3*^*1*^, *red / TM3*, *Sb*, *Ser* (Bloomington stock number 5872)
4. *mle*^*9*^ */ CyO* (Bloomington stock number 5873)
5. *yw*; *CyO / If*
6. *yw*; *TM3*, *Sb / E93*^*4*^

To test for genetic interactions between *H3*.*3B*^*K27R*^ and Polycomb or Trithorax group genes (**Fig 6**), females of the genotype *H3*.*3B*^*K27R*^*/FM7-GFP* or *H3*.*3B*^*K27R*^*/FM7-GFP; tg-H3*.*3B*^*WT*^ were crossed to males of the following genotypes (with origin):

1. *esc*^*5*^, *E(Pc)*^*1*^ */ SM5* (Bloomington Stock Center number 3142)
2. w*; Su(z)12^*4*^, *FRT2A*, *e / TM6C*, *Sb* (Bloomington Stock Center number 24469)
3. w*; FRT82B, *Abd-B*^*Mcp-1*^, *Sce*^*1*^ */ TM6C*, *Sb*, *Tb* (Bloomington Stock Center number 24618)
4. *yw; Pc*^*15*^, *FRT2A / TM3*, *Sb* (Bloomington Stock Center number 24468)
5. *yw; E(z)*^*731*^, *FRT2A / TM3*, *Sb* (Bloomington Stock Center number 24470)
6. *E(z)*^*63*^ */ TM3*, *Sb* (gift of Judy Kassis)
7. *yw; FRT42D*, *Df(2R)Su(z)2-1*.*b8* / SM6b (Bloomington Stock Center number 24467)
8. *brm*^*2*^, *e*, *ca / TM6B*, *Sb*, *Tb*, *ca* (Bloomington Stock Center number 3619)
9. *brm*^*2*^, *trx*^*E2*^, *ca / TM6B*, *Tb*, *ca* (Bloomington Stock Center number 3622)
10. *red*, *e*, *ash2*^*1*^ */ TM6B*, *Tb* (Bloomington Stock Center number 4584)
11. *w; PBac–Utx*^*f01321*^ */ CyO* (Bloomington Stock Center number 18425)
12. *y; FRT82B*, *trx*^*E2*^ */ TM6C*, *Sb*, *Tb* (Bloomington Stock Center number 24160)

Other Genotypes used (with origin):

*H3*.*3B*^*0*^ (gift of Kami Ahmad)

*H3*.*3A*^*2x1*^ (gift of Kami Ahmad)

*Df(2L)BSC110 / CyO* (Bloomington stock number 8835)

### Western blotting

Protein extracts from male *H3*.*3B*^*K27R*^ or *yw* 16–20 hour embryos were prepared as described [33]. Nuclei from male *H3*.*3B*^*K27R*^ or *yw* pharate adults (96–108 hours after puparium formation) were isolated as described [47] with the following modification: nuclei pellets were resuspended in 10mM HEPES_KOH, pH7.9, 2.5mM spermidine, 10mM KCl, 0.1% Triton-X100. Samples were lysed by boiling in Laemmli SDS-PAGE loading buffer and clarified by centrifugation. Proteins were fractionated on BioRad TGX precast Any kD (BioRad 456–9035) gels and were transferred to 0.2um nitrocellulose membranes (BioRad 162–0112) at 100V for 10 minutes then 60V for 20 minutes. Western blot analysis was performed using HRP conjugated secondary antibodies (1:10000 goat anti-Mouse-HRP, ThermoFisher #31430; 1:10000 donkey anti-Rabbit-HRP, GE Healthcare #NA934V) and detected using Amersham ECL prime detection kit (GE healthcare, RPN2232). We used the following antibodies: anti-H3 (1:60000; AbCam AB1791), anti-H3K27me3 (1:1000; AbCam Ab6002), anti-H3K27Ac (1:1000; Active Motif 39135), anti-H3K27me2 (1:1000; Cell Signaling Technologies D18C8), anti-H4K16ac (1:1000; Active Motif 39167), and anti-tubulin (1:30000; Sigma T6074). Western blot signals were quantified with ImageQuant (Amersham) using images captured by Amersham Imager 600. Histone modifications were normalized to corresponding H3 or tubulin signals as indicated. Quantification of signals was based on at least two independent western blots, and each genotype was tested with two quantities of lysate.

### RNA-seq sample preparation and analysis

Total RNA was collected as described previously [42]. Briefly, 30 whole pharate adult males were dissected out of the pupal case and homogenized in 1mL of Trizol, and RNA was extracted according to manufacturer’s recommendation. Qiagen RNeasy columns were used for DNaseI digestion and RNA clean-up. 2ul of 1:1000 diluted ERCC spike-ins were combined with 100ng of total RNA, and then the NuGen Ovation RNA-seq library prep kit was used to generate high-throughput sequencing libraries following to the manufacturer’s protocol. Libraries were sequenced using an Illumina MiSeq. For sequence alignment, modified dm3 fasta and gtf files were generated in which all sequences and annotations for the replication-dependent histone genes were removed and an additional, 5kb chromosome was appended that contained a single, annotated copy of each gene. Individual ERCC spike-in sequences were also appended as individual chromosomes. Genome files for use with the STAR aligner [76] were then generated using parameters:–sjdbOverhang 49. Sequencing reads were aligned using parameters:—alignIntronMax 50000—alignMatesGapMax 50000. Subread [77] was used to count reads mapping to features. DESeq2 [78] was used to identify differentially expressed genes using the lfcShrink function to shrink log-fold changes. Differentially expressed genes were defined as genes with an adjusted p value less than 0.01 and an absolute log2 fold change greater than 1. RPKM values were calculated using a custom R script, which is available upon request. Box plots display the 25^th^ and 75^th^ percentiles, and whiskers depict 1.5 times the inner quartile range. Outliers were removed for clarity. RNA-seq data from H4K16 mutant male third instar wing discs obtained from [45]. Data are publicly available on GEO, accession number GSE117703.

### Microscopy

Adult cuticles were mounted on glass slides in Hoyers and imaged using a digital camera mounted on a stereo-dissecting scope. Wing imaginal disc immunostaining and confocal microscopy was performed as previously described [42]. Mouse anti-Ubx (Developmental Studies Hybridoma Bank) was used at 1:30 dilution.

## Supporting information

S1 Fig (**A**) Bar plot of H3.3B and H3.3A RNA-seq signal from S2 cells treated with RNAi’s targeting dosage compensation components *Msl-2* (blue) and *MOF* (orange) (46). X-linked *H3*.*3B* decreases in both RNAi treatments, relative to control RNAi, whereas autosomal *H3*.*3A* does not decrease. (**B**) Browser shots of RNA-seq reads aligning to the *H3*.*3A* (left) and *H3*.*3B* (right) genes from wild type, *H3*.*3B*^*K27R*^, and double *H3*.*3*^*K27R*^ pharate adult males. Nucleotides in reads matching the wild type reference genome sequence are colored gray. Mismatched nucleotides in reads are indicated in color. The lysine 27 codon has been mutated to arginine, as indicated. (**C**) Plots of observed versus predicted RNA-seq signals for ERCC spike-in control RNAs from wild type, *H3*.*3B*^*K27R*^, and double *H3*.*3*^*K27R*^ pharate adult males.
(TIF)

S1 Table List of genes that are differentially expressed in *H3*.*3B*^*K27R*^ pharate adult males relative to wild type pharate adult males. Genes highlighted in red indicate those that are also differentially expressed in the *H3*.*3*^*K27R*^ double mutants relative to wild type.
(XLSX)

S2 Table List of genes that are differentially expressed in *H3*.*3*^*K27R*^ double mutant pharate adult males relative to wild type pharate adult males. Genes highlighted in red indicate those that are also differentially expressed in *H3*.*3B*^*K27R*^ mutants relative to wild type.
(XLSX)

S3 Table List of RPKM values for all genes from each RNA-seq replicate. (XLSX)

S1 Data Spreadsheet of numerical data used to generate all graphs. (XLSX)
