# Supplementary figures and images for: Lysine 27 of replication-independent histone H3.3 is required for Polycomb target gene silencing but not for gene activation
Source: PLoS Genet. 2019 Jan 30;15(1):e1007932. doi: 10.1371/journal.pgen.1007932 (PMC6370247; doi:10.1371/journal.pgen.1007932)

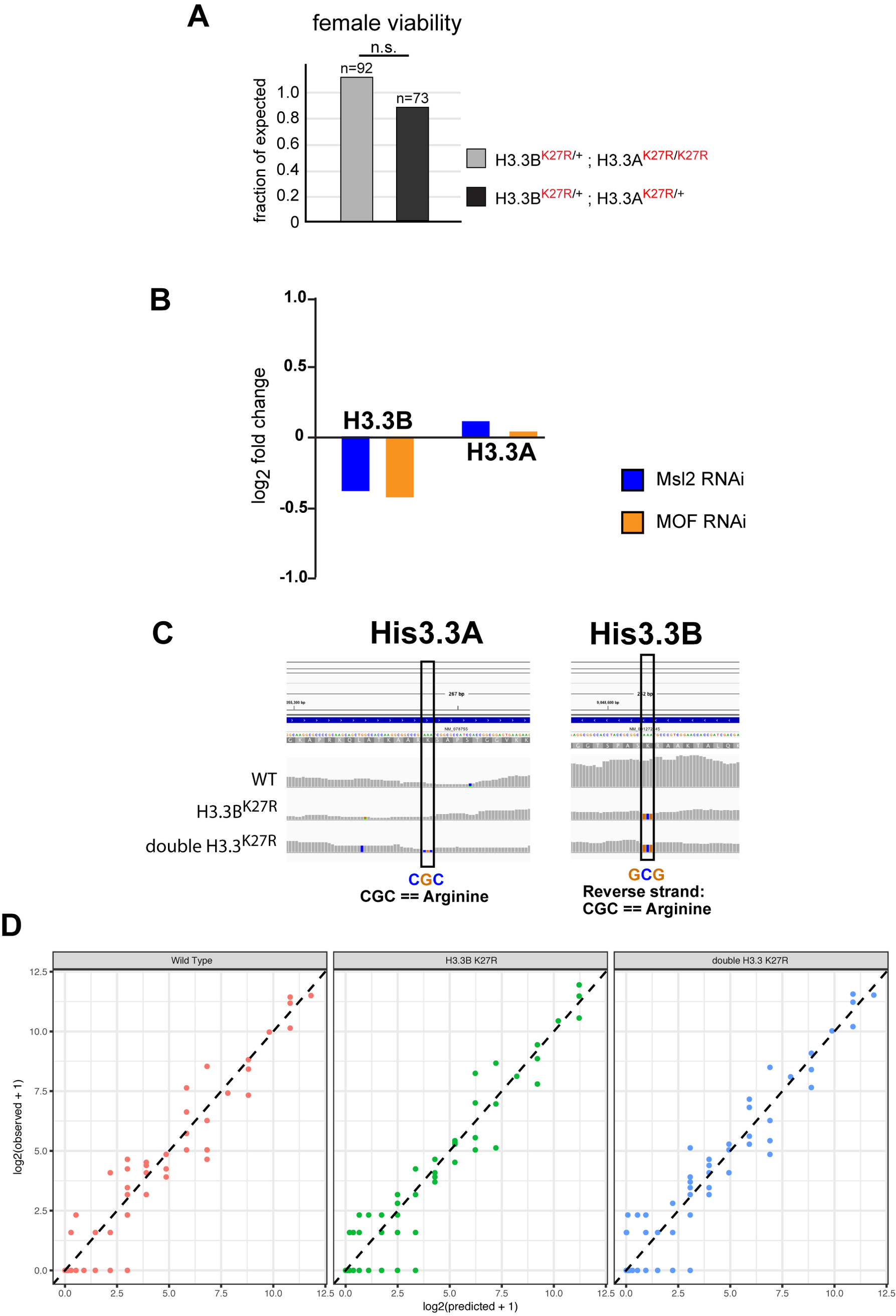

Supplement: S1 Fig — (A) Bar plot of H3.3B and H3.3A RNA-seq signal from S2 cells treated with RNAi’s targeting dosage compensation components Msl-2 (blue) and MOF (orange) (46). X-linked H3.3B decreases in both RNAi treatments, relative to control RNAi, whereas autosomal H3.3A does not decrease. (B) Browser shots of RNA-seq reads aligning to the H3.3A (left) and H3.3B (right) genes from wild type, H3.3BK27R, and double H3.3K27R pharate adult males. Nucleotides in reads matching the wild type reference genome sequence are colored gray. Mismatched nucleotides in reads are indicated in color. The lysine 27 codon has been mutated to arginine, as indicated. (C) Plots of observed versus predicted RNA-seq signals for ERCC spike-in control RNAs from wild type, H3.3BK27R, and double H3.3K27R pharate adult males. (TIF) [file pgen.1007932.s001.tif]
